# Rapid Diagnosis of Central Nervous System Scedosporiosis by Specific Quantitative Polymerase Chain Reaction Applied to Formalin-Fixed, Paraffin-Embedded Tissue

**DOI:** 10.3390/jof8010019

**Published:** 2021-12-27

**Authors:** Robert J. Lauerer, Emely Rosenow, Rudi Beschorner, Johann-Martin Hempel, Georgios Naros, Anna Hofmann, Katharina Berger, Jennifer Sartor-Pfeiffer, Annerose Mengel, Ulf Ziemann, Volker Rickerts, Katharina Feil

**Affiliations:** 1 Centre for Neurovascular Diseases Tübingen, ZNET, University of Tübingen, 72076 Tübingen, Germany; Anna.Hofmann@med.uni-tuebingen.de (A.H.); Katharina.Berger@med.uni-tuebingen.de (K.B.); jennifer.sartor-pfeiffer@med.uni-tuebingen.de (J.S.-P.); annerose.mengel@med.uni-tuebingen.de (A.M.); ulf.ziemann@med.uni-tuebingen.de (U.Z.); Katharina.Feil@uni-tuebingen.de (K.F.); 2 Department of Neurology & Epileptology, University of Tübingen, 72076 Tübingen, Germany; 3 Hertie Institute for Clinical Brain Research, University of Tübingen, 72076 Tübingen, Germany; 4 Mycotic and Parasitic Agents and Mycobacteria, Department of Infectious Diseases, Robert Koch Institute (RKI), 13353 Berlin, Germany; Rosenowe@rki.de (E.R.); RickertsV@rki.de (V.R.); 5 Department of Neuropathology, University of Tübingen, 72076 Tübingen, Germany; rudi.beschorner@med.uni-tuebingen.de; 6 Department of Neuroradiology, University of Tübingen, 72076 Tübingen, Germany; johann-martin.hempel@med.uni-tuebingen.de; 7 Department of Neurosurgery, University of Tübingen, 72076 Tübingen, Germany; georgios.naros@med.uni-tuebingen.de; 8 Department of Neurology & Neurodegeneration, University of Tübingen, 72076 Tübingen, Germany; 9 Department of Neurology & Stroke, University of Tübingen, 72076 Tübingen, Germany

**Keywords:** *Scedosporium apiospermum*, brain abscess, specific qPCR

## Abstract

*Scedosporium (S.) apiospermum* is a typical mold causing cerebral abscesses, often after near-drowning. Infections are associated with high morbidity and mortality due to diagnostic challenges including the need for prolonged incubation of cultures. In addition, histopathological differentiation from other filamentous fungi, including *Aspergillus fumigatus,* may not be possible, excluding early specific diagnosis and targeted therapy. Polymerase chain reaction (PCR) on tissue samples can rapidly identify fungi, leading to an earlier adequate treatment. Due to an extensive spectrum of causative fungi, broad-range PCRs with amplicon sequencing have been endorsed as the best DNA amplification strategy. We herein describe a case with brain abscesses due to *S. apiospermum* in a 66-year-old immunocompromised female patient. While broad-range PCR failed to identify a fungal pathogen from a cerebral biopsy demonstrating hyaline mold hyphae, specific quantitative PCR (qPCR) identified *Scedosporium* and ruled out *Aspergillus*, the most prevalent agent of central nervous system mold infection. A panel of specific qPCR assays, guided by the morphology of fungal elements in tissue or as a multiplex assay, may be a successful molecular approach to identify fungal agents of brain abscesses. This also applies in the presence of negative broad-range fungal PCR, therefore providing diagnostic and therapeutic potential for early specific management and improvement of patient clinical outcome.

## 1. Introduction

An intracerebral brain abscess is a dynamic form of intracranial central nervous system (CNS) infection, defined as a local infection of the brain slowly developing into an encapsulated collection of pus with a connective tissue capsule presenting a mass-like lesion [1].

The growing use of immunosuppressive therapies and organ transplantation have caused an increase in the incidence of cerebral fungal infections. However, occurrence in immunocompetent hosts is also possible. The annual incidence of intracerebral brain abscess is 0.3–1.9/100,000 [2,3,4]. Even though the introduction of broad-spectrum antibiotics and antimycotic medications, improved imaging technology, and intensive care facilities have significantly improved clinical outcome [5], intracranial brain abscesses, especially in immunocompromised patients, remain a problematic complication [2,5].

While mixed bacterial infections are common, fungal pathogens are identified infrequently [5]. Confirmation of the diagnosis and identification of the pathogenic germ without surgical intervention is challenging in intracerebral abscesses, as blood and cerebrospinal fluid (CSF) cultures often remain negative [5].

Hyaline molds, including *Aspergillus (A.) fumigatus* and *Scedosporium (S.)* species pluralis (spp.), are among the most common molds causing fungal brain abscesses. Diagnosis of brain abscesses caused by *S. apiospermum* is difficult due to the slow growth of this mold and non-specific tissue morphology, demonstrating hyaline hyphae. This may impair specific management, leading to high mortality of up to 74% [6,7,8]. In addition, these diagnostic issues may cause underreporting of *S. apiospermum* infections.

While sole cutaneous infections are a potential manifestation of scedosporiosis, systemic infections often happen in patients with immunosuppression or immunocompetent patients who have had a recent history of a near-drowning event [8,9,10,11,12]. Typically, fungal pneumonia leads to the dissemination of the pathogen into the body, which often results in the formation of uni- or multilocular intracerebral brain abscesses [12,13,14]. Due to a broad resistance to antimycotic drugs, treatment of scedosporiosis remains difficult, explaining the unfavorable prognosis in these patients even under the currently-agreed-upon first-line treatment with Voriconazole [6]. Given the scarcity of fungal elements in brain biopsies, the histological discrimination to more common pathogenic fungi such as *Aspergillus* spp. is impaired, and the diagnosis of scedosporiosis has mostly been based on fungal culture, often delaying proper treatment of infected patients [9,14]. In the context of emerging azole resistance in *Aspergillus* and the development of new antimycotic drugs for scedosporiosis, a reliable identification of the pathogen becomes strategically important.

Broad-range fungal PCR with sequencing has been proposed as a reliable approach to identify causative fungal pathogens from formalin-fixed, paraffin-embedded (FFPE) tissue samples [15]. However, typically small brain biopsies with scant fungal elements impose challenges to PCR amplification by broad-range PCRs due to the long amplicons needed for species identification and the increased potential for contamination when using broad-range primers. In addition, sequencing of amplicons used to identify fungal DNA is time-consuming, leading to delayed diagnosis. In contrast, specific quantitative PCR (qPCR) targeting likely pathogens as suggested by the morphology of fungal elements in tissue or as multiplex assay may offer rapid and sensitive fungal identification, leading to better patient outcomes.

Here, we present the case of an intracranial brain abscess caused by *S. apiospermum* in a 66-year-old immunocompromised female patient, in whom broad-range PCR from FFPE was non-diagnostic, but DNA amplification confirmed scedosporiosis and ruled out aspergillosis by the application of two specific qPCR assays on FFPE tissue samples of the cerebral abscess showing hyaline hyphae.

## 2. Case Presentation

A 66-year-old female patient with a history of cryoglobulinemic vasculitis and Sjögren’s syndrome for 36 years initially presented to a peripheral hospital with dry cough, dyspnea, and a general feeling of weakness for some days before admission. Body temperature at admission was 37.7 °C.

**Figure 1:**
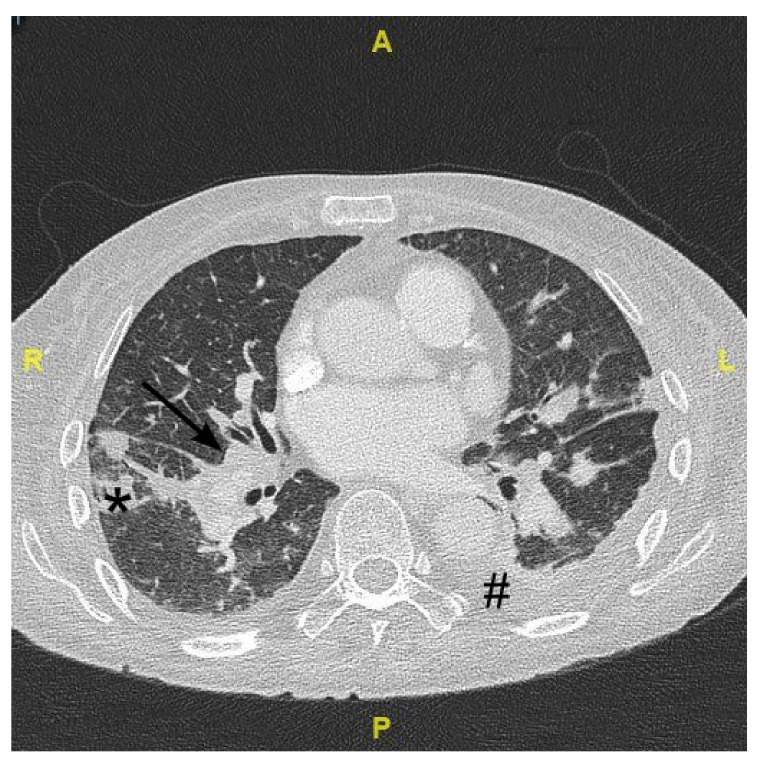
Cropped axial computed tomography image of the thorax on day 36 after admission showing mediastinal and bilateral hilar lymphadenopathy (arrow) as well as focal inflammatory consolidations within the lung parenchyma (star). Additionally, there are small pleural effusion (hash) and accompanying dystelectatic pulmonary areas. Abbreviations: A anterior; P posterior; R right; L left.

Chest X-ray showed increased opacity in the right lower lung field. A computed tomography (CT) scan of the thorax revealed atypical pneumonia and mediastinal lymphadenopathy (see Figure 1). Laboratory results on admission showed pancytopenia (white blood cell count 1100/µL; neutrophilic granulocytes 780/µL; lymphocytes 0.07/µL; eosinophilic granulocytes 0/µL; basophilic granulocytes 0/µL; hemoglobin 6.7 g/dL; thrombocytes 133,000/µL), increased levels of C-reactive protein (CRP) 23 mg/dL and low blood albumin of 1.9 g/dL. On admission, the patient had been on immunosuppressive treatment with Methotrexate (MTX) 20 mg/week and Methylprednisolone 5 mg/day. MTX had been started approximately two years before the current admission and had previously been stopped due to leukopenia. Before that, she had been treated with Prednisolone and Hydroxychloroquine for several years. As she had had an allergic reaction to penicillin in the past, antibiotic treatment with Meropenem and Clarithromycin was started for suspected bacterial pneumonia.

Nine days after admission, the patient developed an acute respiratory emergency and required intubation and intensive care treatment. A CT scan revealed pulmonary embolism, multiple pulmonary nodules, and mediastinal lymphadenitis. An anticoagulatory treatment with Enoxaparin in a weight-adjusted dose was initiated. Microbiologic cultures from bronchoalveolar lavage (BAL) did not grow microorganisms and thus failed to identify potential agents causing pneumonia in our patient. As the antibiotic treatment was evaluated as ineffective, empiric oral Voriconazole treatment with 400 mg/day was started on day 11 after admission for suspected aspergillosis, and antibacterial treatment was changed to Meropenem and Linezolid.

On day 22, after hospital admission, the patient was found to be somnolent. CT scan and magnetic resonance imaging (MRI) of the brain showed multiple abscess formations in the left *lentiform nucleus* and the periventricular region with meningeal contrast enhancement in T1-Sequences. CSF showed mild pleocytosis with 9 cells/µL (reference < 5 cells/µL) and an increased protein level of 49 mg/dL (reference 0–45 mg/dL). Externally acquired follow-up MRI on day 34 additionally showed intraventricular fluid levels.

A CT scan of the abdomen on day 23 after admission revealed splenomegaly and enlarged periaortic lymph nodes. Furthermore, transesophageal echocardiography (TEE) on the same day and cardio MRI on day 26 after admission showed a thrombus 6.5 cm × 1.2 cm in the right atrium. Blood cultures remained negative. Modified Duke criteria for endocarditis were not fulfilled [16].

**Figure 2:**
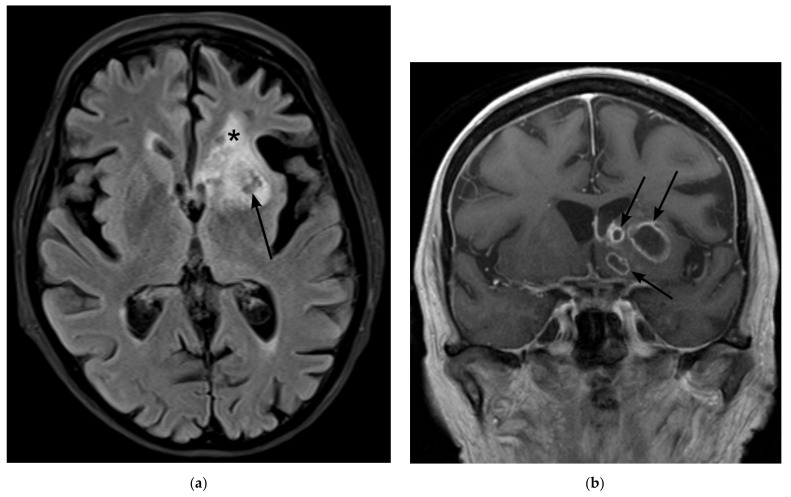

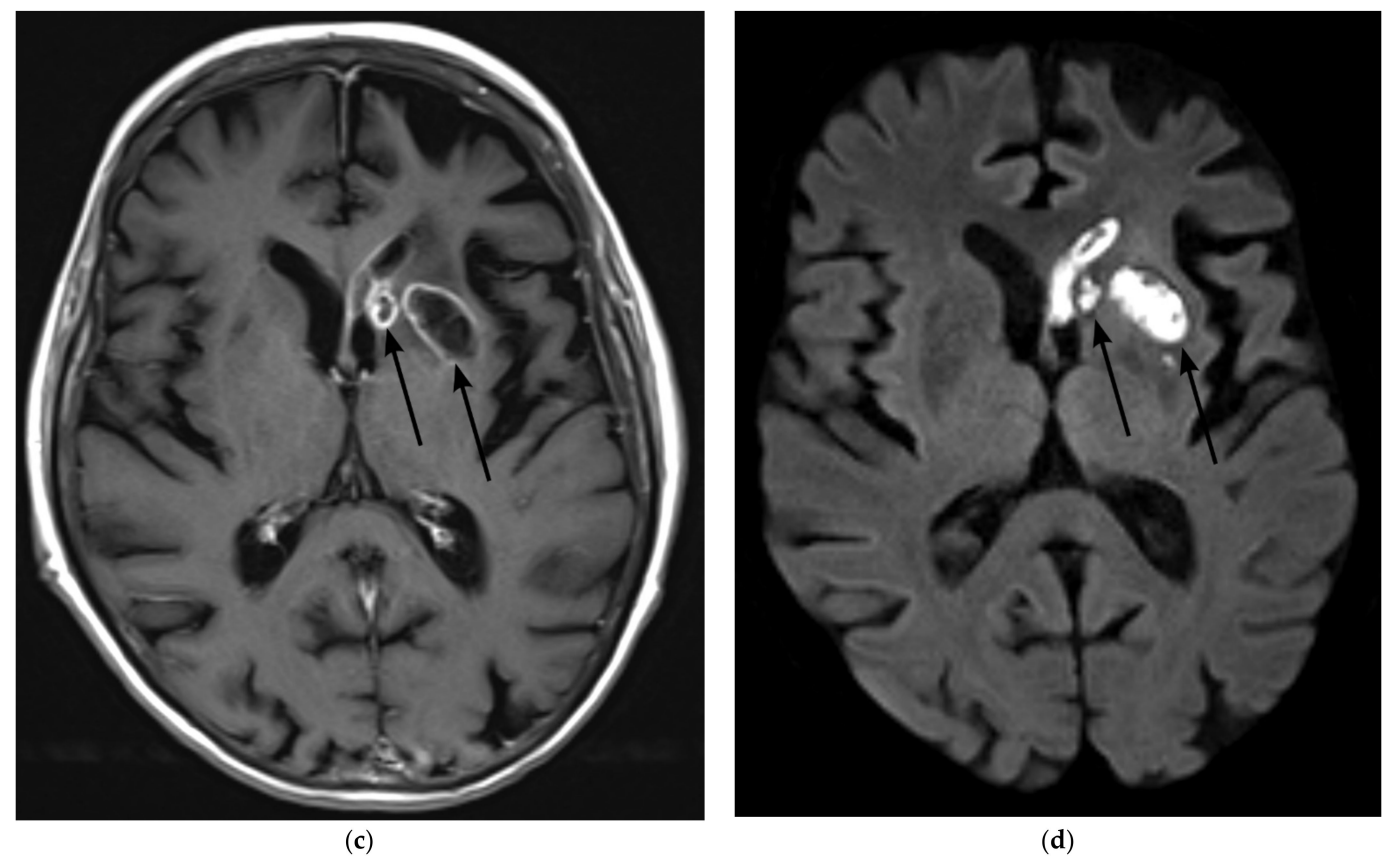
Magnetic Resonance Imaging (MRI) on day 37 after initial admission to the hospital (**a**) Fluid attenuated inversion recovery (FLAIR) shows lesions with mixed T2 signal (central hypointense areas with perifocal hyperintense rim) (arrow) and with surrounding edema indicated by laminar T2-hyperintense signal (star) at the anterior horn of the left lateral ventricle. (**b**,**c**) Contrast-enhanced T1-weighted image in coronal (**b**) and axial (**c**) angulation show multiple centrally hypointense ovoid lesions with rim-enhancement (arrows) (**d**) b-1000 image of diffusion-weighted imaging shows restricted diffusion within the lesions (arrows), indicating an abscess.

On day 34, after initial hospital admission, the patient was referred to the university hospital of Tübingen and admitted to the intensive care unit. CT of the thorax on day 36 showed persistent pulmonary embolism and multiple aneurysms of the pulmonary arteries that were considered due to fungal infection (Figure 1). Follow-up MRI on day 37 showed a consistent abscess formation near the left lateral ventricle (Figure 2). Microbiological cultures from bronchoalveolar lavage on day 38 remained negative. Voriconazole treatment was continued until day 43, when the patient was transferred to a different ward, where antimycotic treatment was discontinued as Aspergillus and Candida antigen tests from serum on days 42 and 49 were negative. Multiple CSF microscopy and blood culture samples remained negative. On day 49, empiric treatment with Caspofungin was started.

No final diagnosis could be made regarding the generalized lymphadenopathy of the patient. Bone marrow samples on day 42 and lymph node biopsy on day 44 showed no clear evidence for lymphoma. However, angioblastic T-cell lymphoma was suspected as there was B- and T-cell clonality.

**Figure 3:**
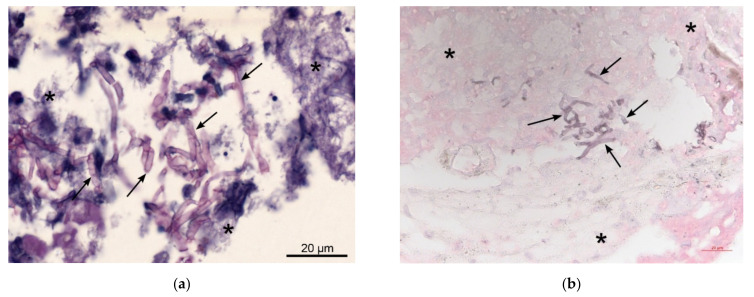
Pathological images of brain biopsy (**a**) The brain biopsy shows necrotic brain tissue (star) with mostly narrow, septated hyaline mold hyphae (arrow) without specific branching pattern or conidiation in tissue, suggesting hyalohyphomycosis without characteristic findings indicative of aspergillosis or scedosporiosis. Periodic acid–Schiff (PAS) staining; (**b**) Grocott’s Methenamine silver staining.

Brain biopsy on day 52 after initial hospital admission showed reactive astrogliosis, cellular infiltrates with lymphocytes, granulocytes, and plasma cells with necrosis and narrow hyaline hyphae that were interpreted as evidence for aspergillosis (Figure 3). Quantitative PCR assays applied to the FFPE brain tissue amplified DNA of *S.* and did not amplify DNA of *Aspergillus*. Mycotic cultures of the specimen subsequently grew *S. apiospermum*, as identified by partial sequencing of the Beta-Tubulin gene. In vitro resistance (CLSI M38) testing demonstrated good activity of Voriconazole (minimal inhibitory concentration (MIC) 1 µg/mL), moderate activity of Anidulafungin (MIC 4 µg/mL), and less activity of Amphotericin B, Posaconazole, Itraconazole, and Isavuconazole (MIC each 8–16 µg/mL).

Treatment with Voriconazole was reinitiated on day 55 after detection of *S. apospermium* in the brain tissue sample. Dosage was adjusted according to serum levels. Additional PCR from a blood sample drawn on day 72 after initial admission could not detect *S. apospermium* DNA.

**Figure 4:**
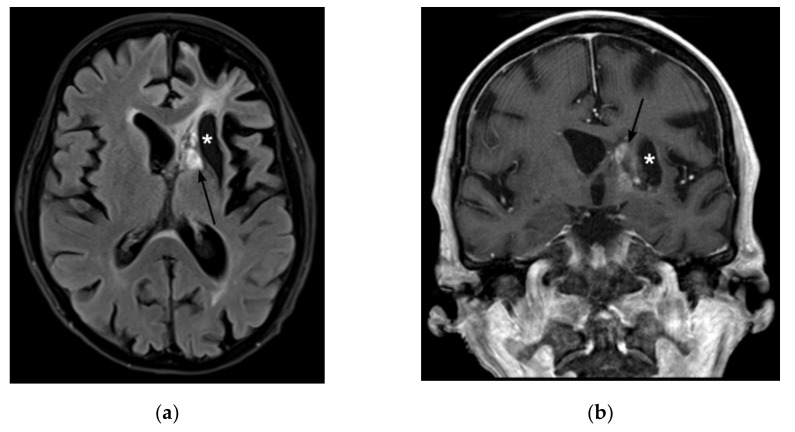
Magnetic Resonance Imaging (MRI) on day 251 after initial admission to the hospital (**a**) Fluid attenuated inversion recovery (FLAIR) shows regressing lesions with mixed T2 signal (arrow) and a hypointense tissue defect at the former site of the abscesses (star). (**b**) Contrast-enhanced T1-weighted image in corona shows regressive enhancement of the lesions.

To this time point, the patient was under treatment with Voriconazole and Caspofungin. Ninety-seven days after the initial hospital admission, the patient’s neurological condition had improved, and she could be transferred to a rehabilitation hospital, even though dysarthria was still present and the patient was still not fully oriented. Follow-up MRI scans at day 251 after initial hospital admission showed a decrease on the abscess with focal ventriculitis still present (Figure 4). While still under antimycotic treatment, Scedosporium qPCR from CSF and serum samples taken on day 252 remained negative. Up to the day of submission of this case report at day 290, the patient is still under treatment with Voriconazol and her neurological condition has improved further.

## 3. Materials and Methods

DNA extraction was performed from FFPE tissue, as reported previously [17]. In short, the formalin-fixed, paraffin-embedded tissue block was cut into 5 µm slices. Three slices were used for DNA extraction after Paraffin removal using octane. We used the MasterPure™ yeast DNA Purification Kit (LGC Lucigen, Middleton, WI, USA) according to the manufacturer’s instructions with two modifications. First, we added a bead beating using Silicon-carbide sharp particles (BioSpec Products Inc., Bartlesville, OK, USA) in a FastPrep-24™5G (MP Biomedicals, LLC, Solon, OH, USA) at 5 m/s for 60 s. Second, we added a 3 h heating step at 90 °C followed by 5 min on ice. Fungal DNA was amplified using a broad-range assay targeting the 28S rRNA Gene (primer 10f–12r) as described previously with amplicon sequencing using Sanger sequencing [17,18]. In short, the primers 28S 10f and 12r amplify a 330–350 bp sequence of a conserved region of the 28S rRNA gene using 5 µL of extracted DNA (diluted in 75 µL) and 45 µL master mix including SYBR green for amplicon detection and melt curve analysis in an Applied Biosystems™ 7500 Real-Time PCR System (Thermo Fisher Scientific Inc., Foster City, CA, USA). The master mix consisted of 20 µL SYBR™ select master mix (Thermo Fisher Scientific Inc., Carlsbad, CA USA), 400 nM of forward and reverse primer, 0.002% Triton^TM^ X-100 (Sigma-Aldrich, Saint Louis, MO, USA), and 20.9 µL Milli-Q^®^ water. The PCR cycling conditions consisted of a 2 min Uracil N-glycosylase activation at 50 °C following a pre-melt for 10 min at 95 °C and 45 cycles of 15 s melting at 95 °C, 30 s annealing at 55 °C, and 40 s extension at 72 °C. The melt curve was performed beginning with a 10 s step at 95 °C, followed by 5 s at 65 °C and 50 s at 95 °C. Positive broad-range PCR was defined as amplification of DNA of identical melt curves in two technical replicates. Contamination was ruled out by negative extraction and master-mix controls. *Aspergillus* DNA was amplified using a specific qPCR using TaqMan^TM^ chemistry targeting the 18S rRNA Gene of *Aspergillus* with an amplicon length of 114 bp. The forward primer 5′-GATAACGAACGAGACCTCGG-3′, reverse primer 5′-AGACCTGTTATTGCCGCGCGC-3′, and probe 5′-[FAM]-CTTAAATAGCCCGG –[MGBEQ]-3′ were used [19]. Each 25 µL reaction contained 5 µL DNA and 20 µL master mix consisting of 800 nM forward and reverse primer, 200 nM probe, and 1× GeneAmp™ PCR Gold Buffer, 2.2 U AmpliTaq^®^ Gold DNA Polymerase, 0.05 U AmpErase^®^ Uracil N-glycosylase (UNG), 1.0 mM GeneAmp™ dNTP Blend with dUTP and 6 mM MgCl_2_ (all from Applied Biosystems, Carlsbad, CA, USA), 0.002% Triton^TM^ X-100 (Sigma-Aldrich, Saint Louis, MO, USA), and 6.91 µL Milli-Q^®^ water. This PCR assay was run on an Applied Biosystems™ 7500 Real-Time PCR System (Thermo Fisher Scientific Inc., Carlsbad, CA, USA). The PCR was initialized with a 2 min Uracil N-glycosylase activation at 50 °C following a pre-melt for 10 min at 95 °C and 45 cycles of 15 s melting at 95 °C and 65 s annealing and extension at 65 °C. *Scedosporium* DNA was amplified using a specific qPCR using TaqMan^TM^ chemistry targeting the ITS2 region of *Scedosporium* with an amplicon length of 255 bp. The specific forward primer 5′-GAGCGTCATTTCAACCCTCG-3′, a panfungal reverse primer 5′-ATATGCTTAAGTTCAGCGGGT-3′, and a *Scedosporium* specific probe 5′-[Cy5]-TCGCATTGGGTCCCGGCGGA-[BBQ]-3′ were used [20]. One reaction contained 5 µL DNA and 20 µL master mix consisting of 350 nM forward and reverse primer, 250 nM probe, 10 µL TaqMan^TM^ Universal Master Mix II, with UNG (Thermo Fisher Scientific Inc., USA), 0.002% Triton^TM^ X-100 (Sigma-Aldrich, Saint Louis, MO, USA), and 8.14 µL Milli-Q^®^ water. This PCR assay was performed on a Bio-Rad CFX96 Touch Real-Time PCR Detection System. The qPCR was initialized by 10 min at 40 °C following a 10 min pre-melt at 94 °C and 45 cycles of 10 sec melting at 94 °C and 60 s annealing and extension at 60 °C. The TaqMan qPCRs were defined as positive for amplicon detection in two technical replicates, no detection in extraction, and no-template controls, as well as no inhibition of the reaction.

Successful DNA extraction was documented by a specific qPCR targeting the human 18S rRNA gene. Inhibition of the PCR reaction was ruled out by an internal amplification control [18]. Possible contaminations were monitored with no-template controls for all qPCR assays. All primers and probes were purchased from Eurofins Genomics (Germany).

## 4. Discussion

We herein describe a case of scedosporiosis in an immunocompromised patient with no history of a near-drowning event. Histopathological examination could not identify scedosporiosis in the first place, as the microscopic discrimination with other fungi such as *Aspergillus* spp. can be difficult, especially in small tissue samples such as brain biopsies [12]. Genus-specific qPCR assays from the brain tissue provided a fast diagnosis of scedosporiosis. Furthermore, qPCR assays ruled out the presence of the most common mold causing hyalohyphomycosis, *A. fumigatus*, providing a basis for rapid, specific patient management.

However, in the cases described in the literature, the diagnosis of brain abscesses by *S. apiospermum* has mostly been based on brain biopsy followed by cultivation of the aspirate, microscopy and molecular identification of the isolate using PCR [8,9] or sequencing of tissue material [13], although in some cases, extracerebral infection sites revealed scedosporiosis, e.g., in BAL cultures [21]. In many cases, a fungal infection can be diagnosed based on histopathology, but the identification of species is often only achieved by PCR or sequencing. An overview of multiple case reports on the diagnosis of *S. apiospermum* in brain abscesses is given in Table 1.

Experts’ opinion concluded that broad-range PCR with amplicon sequencing is the preferred approach to identify fungi at the genus level from FFPE tissue specimens showing fungal elements. While in our case, broad-range PCR was negative, specific TaqMan^TM^ qPCRs selected by histopathology allowed for a rapid diagnosis of scedosporiosis. This suggests that specific PCRs might be successfully applied in a subsequent manner guided by histopathology or as a multiplex assay for fungal identification even in the absence of a diagnostic broad-range PCR. Differences in amplicon length or difficulties in sequencing due to contaminating fungal DNA may impair the broad-range approach. In addition, this approach may accelerate diagnostic process, as sequencing of broad-range amplicons is not necessary. In this reported case, a differentiation of *L. prolificans* would have been useful but was not available. A PCR targeting *Fusarium* would have been a logical next assay in hyalohyphomycosis but was not performed as cultures grew *S. apiospermum.* It has been demonstrated before that specific qPCR assays offer improved sensitivity compared to broad-range PCR and sequencing when applied to FFPE tissue in the context of mold infections and histoplasmosis [22,23]. The optimal molecular strategy may include both broad-range assays and specific assays selected for fungi, likely causing a given histopathologic result.

*S. apiospermum* exhibits a low susceptibility to many antimycotic drugs such as Amphotericin B and Nystatin [12,31]. Voriconazole has been identified to be the most effective agent in the treatment of scedosporiosis with mixed evidence on combination therapies with other antimycotic drugs [6,10,12]. However, recently novel antifungals with activity against *Scedosporium,* including Fosmanogepix, Olorofim, and Ibrexafungerp, have entered clinical trials, necessitating the identification of the etiology of fungal infections to guide therapeutic choices [32]. There is only limited evidence for the duration of the treatment regime. In most cases reported, long-term treatment has been applied [6,10,13].

Of note, no near-drowning event was reported, and the patient did not undertake any traveling before infection. We suspect a primary pulmonary infection in this patient in the context of immunosuppression with MTX and prednisolone. Systemic infection appears to have led to endocarditis with multiple septic embolisms and a cardiac thrombus, leading to pulmonary embolism and the formation of the cerebral abscesses after paradoxical embolism. Previously, one patient with *S. apiospermum*-associated generalized lymphadenopathy has been described in the literature [33]. Mediastinal lymphadenopathy has been described in three patients suffering from *S. apiospermum* infection after lung transplantation [34]. Additionally, our patient suffered from low albumin blood levels, a previously described risk factor regarding *S. apiospermum* infection [7]. Fortunately, early treatment with Voriconazole was initiated on day 11 after the first admission, even though no clear evidence of fungal infection was found at that time point. Simultaneously, antibiotic treatment was established, as brain abscesses are more likely to be caused by bacteria than fungi [5]. Brain biopsy was obtained late, as the MRI findings remained stable. With no clear evidence of a fungal infection and negative *Aspergillus* and *Candida* antigen tests, Voriconazole was discontinued and later reintroduced. Furthermore, the typically used *Aspergillus*–Galactomannan antigen assays have moderate sensitivity and high specificity and should therefore not be used to rule out aspergillosis [35]. Likewise, single *Candida* antigen or antibody tests are not able to completely dismiss invasive Candidiasis [36]. Discontinuation of the antifungal therapy could have had a fatal outcome as the lethality of infections with *S. apiospermum* is up to 74%, even when appropriately treated with Voriconazole [6]. Earlier identification of the pathogen could only have been obtained by an earlier brain biopsy and has been commonly advised in the past, as blood culture and CSF samples often cannot contribute to the isolation of the pathogen in cerebral abscesses in general [5]. However, a more conservative approach without surgery might be possible with small abscesses with a known pathogen [37] but is commonly not suggested [38]. With this high lethality of scedosporiosis, we propose an early biopsy of brain abscesses for histopathology, culture, and specific molecular tests in immunocompromised patients to rule out scedosporiosis and empirical treatment with Voriconazole until then.

To conclude, given the difficult diagnosis of scedosporiosis using culture methods, we advocate early brain biopsy in brain abscesses and the identification of *Scedosporium apiospermum* using specific qPCR out of tissue samples to provide early diagnosis and treatment to patients.

## Figures and Tables

**Table 1:** Overview of several case reports describing the diagnosis of *Scedosporium apiospermum* involved in brain abscesses. + positive, (+) indicative of fungal infection, −negative, o not performed or not reported, CSF cerebrospinal fluid, BAL bronchoalveolar lavage, FESS functional endoscopic sinus surgery.

Literature	Biopsy	Histopathology	Culture	Microscopy of Cultured Material	PCR	Sequencing	Comments
Buzina et al., 2006 [8]	+	o	Blood: − BAL:− Biopsy: +	+	Culture: +	Culture: +	
Mursch et al., 2006 [24]	+	o	CSF: − Biopsy: +	o	o	Culture: +	
Caggiano et al., 2011 [9]	+	(+)	Biopsy:+	+	Molecular analysis of culture was performed but not further specified.		
Nakamura et al., 2011 [21]	o	o	BAL: +	BAL: +	BAL: +	BAL: +	
Tammer et al., 2011 [25]	+	(+)	Biopsy: +	+	−	Culture: +	
Henao-Martinez et al., 2013 [26] Case 1	o	o	FESS: +	+	o	o	
Henao-Martinez et al., 2013 [26] Case 2	−/+	(+)	CSF: +	o	o	o	1st Biopsy −
Lin et al., 2013 [27,28]	+	(+)	Biopsy: +	o	o	o	
Wilson et al., 2013	+	(+)	Biopsy: + Sputum: +	+	Biopsy: +	+	
Williams et al., 2016 [29]	+	−	CSF: − Biopsy: +	o	CSF: − Biopsy: +	o	
Signore et al., 2017 [13]	−/+	−/+	Blood: − Biopsy: +	o	Biopsy: − Blood: −	Tissue: +	1st Biopsy −
Lee et al., 2018 [14]	+	o	Sputum:− CSF:− Biopsy:+	o	o	o	
Sudke et al., 2020 [30]	+	+	Culture +	+	o	o	

## Data Availability

The data presented in this study are available on request from the corresponding author. The data are not publicly available due to privacy rights.
